# The Risk Assessment of Pesticide Ingestion with Fruit and Vegetables for Consumer's Health

**DOI:** 10.1155/2021/9990219

**Published:** 2021-06-14

**Authors:** Gordana Jurak, Jasna Bošnir, Domagoj Đikić, Ana Mojsović Ćuić, Iva Pavlinić Prokurica, Aleksandar Racz, Tomislav Jukić, David Stubljar, Andrej Starc

**Affiliations:** ^1^Dr. Andrija Štampar Teaching Institute of Public Health, Zagreb, Croatia; ^2^Department of Biology, Faculty of Science, University of Zagreb, Zagreb, Croatia; ^3^University of Applied Health Sciences, Zagreb, Croatia; ^4^Croatian Agency for Agriculture and Food, Zagreb, Croatia; ^5^Department of Internal Medicine, History of Medicine and Medical Ethics, Faculty of Medicine Josip Juraj Strossmayer, Osijek, Croatia; ^6^Department of Research & Development, In-Medico, Metlika, Slovenia; ^7^Institute of Microbiology and Immunology Faculty of Medicine Ljubljana, Ljubljana, Slovenia; ^8^Faculty of Health Sciences, University of Ljubljana, Ljubljana, Slovenia

## Abstract

Pesticides are chemicals used in agriculture to protect crops from pests. In addition to protection during cultivation, they are also used after harvesting to extend the shelf life of products. Postharvest control stands out, especially when it comes to products imported from distant countries, resulting in increased concentration of pesticides and risk to human health consuming such products. In this study, analyses of pesticide residues were performed on 200 samples of fruits and vegetables. Pesticide residues were identified and quantified in 30 out of 200 samples. Study results revealed imazalil to be the most frequently detected pesticide. Risk assessment was performed on the obtained results, and it was carried out separately for adults and for children under 6 years of age. Imazalil showed the highest ARfD percentage for adults (max% ARfD 251%), and these values were especially high on risk assessment for children, where they amounted up to max% ARfD 1087%. The study of imazalil impact was performed on 16 Swiss albino mice divided into two groups and 4 subgroups. Experimental group animals were treated with the corresponding NOAEL dose of imazalil (10 mg/kg) for 28 days. Body weight was measured before each pesticide application on a digital electronic Sartorius scale. Peripheral blood analysis was performed after 28-day animal exposure to pesticides. Animals were anesthetized, blood samples were obtained by cardiac puncture, and red blood cell (RBC) count, hemoglobin (Hb) concentration, and white blood cell (WBC) count were determined by standard hematological methods. The organs for determination of imazalil concentration were extracted immediately upon animal sacrifice and stored in a freezer at -80°C until analysis. Results show difference in gain weight, and an increase in WBC count was recorded in the experimental group as compared with a control group of animals. The highest imazalil levels were recorded in adipose tissue (45.2‰) which proves tendency to accumulate.

## 1. Introduction

Croatia belongs to the group of countries with relatively low use of pesticides for soil treatment, which is not the result of the rational pesticide utilization and their strict control, but most likely due to poor agricultural development with very low and nationally inadequate cultivation of agricultural products. Therefore, the Croatian market is overflown with a variety of fruit and vegetables, mostly imported from the European Union countries and other parts of the world. It is a well-known and legally justified fact that fruit and vegetables are treated with various types of pesticides to increase gain and food shelf life. Agricultural products can be treated with a single active substance or with several such substances simultaneously, which results in human exposure to their action and the potential adverse health effects. Therefore, it is logical to pose questions related to the combined intake of several active substances and their adverse effects in the human body. There is not enough information either on the combined action of chemicals or of pesticides, including long-standing professional exposure. Available data indicate that 95% of toxicological studies evaluated exposure to only one chemical [1–3]. A synergistic study of six different pesticides acting upon *Daphnia magna* as a test organism confirmed the theory on the combinations of some pesticides to exert enhanced toxicological activity [4]. A study conducted on honeybees (*Apis mellifera*) and bumblebees (*Bombus terrestris L*) led to similar conclusions [5–7].

Considering residual pesticides in and/or on foodstuff and their safety, a product safe for use in humans is the one that contains particular pesticide in the amount determined by the respective By-law or European Union Directive [8].

The number of active substances as well as the variable solubility and polar characteristics of each individual pesticide has led to the development of various analytical methods for their identification and quantification. Development of multiresidue methods was not simple at all, knowing the very demanding preconditions to be met to enable pesticide quantification in the sample analyzed. Gas chromatography methods using different types of detectors are now available, which can quantify only some of the active pesticide components on the basis of retention times. Besides being quite time-consuming and using large amounts of solvents, these methods produced false-positive results due to interferences from the matrices that coeluted concurrently with the target analyte [9].

In 2003, the Quick, Easy, Cheap, Effective, Rugged, and Safe (QuEChERS) method was introduced and adopted by the analysts as a very rapid, simple, and efficient method [10]. Literature data report high recovery of even 70%-110% associated with this method in routine analytical procedures with the use of small amounts of nonpolar diluents (10 ml) [11–13].

This analytical method has provided insight into the qualitative and the quantitative presence of particular pesticides in fresh fruit and vegetables, while confirming the fact that humans are continuously exposed to particular pesticides to some extent if taking fresh fruit and vegetables [14, 15].

In order to determine the possible adverse effect of pesticides on human health by taking fresh fruit and vegetables, the risk should be assessed for each individual pesticide and for their combinations because it is known that two or more substances found together in the body can assume quite different actions than each of them alone [16].

The first attempts at such a risk assessment were reported as early as the 1970s and referred to vinyl chloride, published by the Environmental Protection Agency (EPA), entitled “Quantitative Risk Assessment Community Exposure to Vinyl Chloride” [17]. Over the years, risk assessment has become increasingly important and various tools for risk assessment have been developed and used by researchers [18–23].

In the present study, the multiresidue QuEChERS method was employed for pesticide isolation. A combined technique of gas chromatography and mass spectrometry (GC-MS) was used to determine the presence and level of particular pesticides in selected fresh fruit and vegetables. The potential risk for human health was assessed by the use of the EFSA software PRIMO 3.1 model. The results thus obtained on the prevalence of particular pesticides in the study samples revealed the most prevalent pesticide, which was then tested on experimental animals.

## 2. Material and Methods

A risk assessment of the obtained results was made.

### 2.1. Preparation of the Fruit and Vegetable Samples by the Use of the QuEChERS Method

A total of 200 samples of fresh fruit and vegetables were analyzed (120 fruit and 80 vegetable samples).

Study samples were divided into three groups according to their characteristics. Both imported and domestic samples were included and analyzed for the presence of pesticides to assess their health safety.

All samples were prepared in the same way, as follows: 1 kg of sample is homogenized and 10 g taken for further procedure. Internal TPP standard and 10 ml ACN, 4 mg MgSO_4_, 1 g NaCl, 1 g trisodium citrate dehydrate, and 0.5 g disodium hydrogen sesquihydrate are added to the sample. The sample thus prepared is centrifuged for 5 min at 3500 rpm [15, 24]. 6 ml of the prepared sample aliquot is taken for further procedure. The previously prepared SPE kit containing 150 mg primary secondary amine (PSA), 150 mg C18, and 900 mg magnesium sulfate is used for the way and fat-containing samples. The SPE kit used for pigmented samples contains 150 mg PSA, 15 mg graphite carbon black (GCB), and 900 mg magnesium sulfate. The SPE kit used for extremely pigmented samples contains 150 mg magnesium sulfate, 44 mg GCB, and magnesium sulfate [15]. The injection volume is 1 *μ*l.

### 2.2. Pesticide Identification and Quantification by the GC-MS Technique

The mass proportion of residual pesticides was determined on a GCMS-QP 2010 Plus equipped with PTV autoinjector model AOC-20i. The Restek capillary column RTX-OPPPesticides (30 m × 0.25 mm i.d.) and film thickness 0.25, with helium (He 6.0) flow of 1.99 ml/min, were employed for separation.

Pesticide identification and quantification were performed by the use of the SIM mode analysis based on one main ion and two confirmation ions and retention time. The concentration is calculated automatically by the use of the GCMS solution software, based on the ratio of the analyte peak surface divided by the standard peak surface.

Recovery: recovery of 70%-120% proposed in the “Analytical Quality Control and Method Validation Procedures for Pesticide Residues Analysis in Food and Feed” was achieved by the use of three inoculated representative samples (Table 1) [25].

Limit of quantification: quantification limit was defined as the lowest inoculated level meeting the method performance to the acceptable criterion (mean recovery for each representative sample ranging from 70% to 120% with RSD < 20%) [25].

### 2.3. Risk Assessment

Risk assessment for acute exposure to pesticides was calculated by the EFSA Pesticide Residue Intake Model-PRIMo model (rev 3.1), using the concentration values presented in the appropriate table and acute reference dose (ARfD) if it was established. Pf (peeling factors) values for citrus fruits and potato are not used in these calculations. Risk assessment was performed separately for adults and for children under 6 years of age. Food is considered safe for consumption if the estimated intake of harmful substances does not exceed the ADI or ARfD (acute reference dose) values. During exposure assessment, except for data on concentrations of residue, consumption data for a particular type of food has also been taken into account, bearing in mind the nutritional habits of a particular population.

### 2.4. Experimental Animals

The study was performed on 16 male and female Swiss albino mice. The animals were obtained from the Department of Animal Physiology, Faculty of Science University of Zagreb, Croatia. The study protocol was approved by the above institution's Ethics Committee. Experiments were carried out in line with the respective guidelines on keeping and using experimental animals [26] and European Union Directive [27].

Animals were fed a standard laboratory diet, tap water *ad libitum*, and received 12 hours of light per day. The standardized diet was 4 RF 21, Mucedola (Settimo Milanese, Italy). The composition of standardized pellet mouse feed included wheat, wheat straw, hazelnut skins, maize, soybean hulled, corn gluten feed, fishmeal, dicalcium phosphate, sodium chloride, whey powder, soybean oil, yeast, contained 12% moisture, 18.5% protein, 3% fats, 6% crude fibers, 7% crude ash, E672 (vitamin A), E671 (vitamin E), E1 (Fe), E2 (I), E3 (Co), E4 (Cu), E5 (Mn), and E6 (Zn). Experimental and control animals were kept under identical conditions.

At initial pesticide application, animals were aged 60 ± 5 days, mean weight 25 g.

Animals (*n* = 16) were divided into control and experimental groups. Each group consisted of 8 mice of both sexes (4 males and 4 females). During 28 days, the control group received pure edible sunflower oil (0.25 ml/25 g body weight per day) by gavage. The experimental group was treated with the corresponding NOAEL dose of imazalil (10 mg/kg) for 28 days [17, 27]. The concentrated pesticide (Sigma, proanalysis, 99.5% purity), was suspended in pure edible sunflower oil before application. The pesticide was administered orally by gavage in the same volume as in the control animals (0.25 ml/25 g body weight per day). All animals in both groups were sacrificed on day 28 of pesticide treatment.

Body weight was measured before each pesticide application on a digital electronic Sartorius scale, with precision of ±0.1 g. Peripheral blood analysis was performed after 28-day animal exposure to pesticides. Animals were anesthetized (Xylapan/Narcetan), blood samples were obtained by cardiac puncture, and red blood cell (RBC) count, hemoglobin (Hb) concentration, and white blood cell (WBC) count were determined by the Beckman Coulter hematological counter. The organs for determination of imazalil concentration were extracted immediately upon animal sacrifice and stored in a freezer at -80°C until analysis.

Organ weight was measured on a torsion balance (Torsion Balance, USA) with precision of ±0.1 mg. Muscle, kidney, adipose tissue, and brain samples from 4 male and 4 female mice were taken for residue pesticide analysis. The organs were pooled (in order to gain enough sample for pesticide extraction since mouse organs in mg weight ratio) and prepared by the QuEChERS method for analysis of imazalil residues by the GC-MS technique and analyzed as triplicate. Upon determination of imazalil concentration in particular organs, the mean concentrations were calculated and expressed as imazalil ‰ of entry dose in the analyzed organs from male and female animals.

## 3. Results

### 3.1. Determination of Residual Pesticide Concentration in Study Samples

Pesticide residues were detected in 31 of 200 (15.5%) fresh fruit and vegetable samples analyzed. In fruit samples, pesticide residues were found in 22 of 120 (18.3%) samples. Imazalil was detected in 18 samples at a concentration range of 0.020-4.1 mg/kg. Chlorpyriphos was found in 8 samples at a concentration ranging from 0.030 mg/kg to 0.27 mg/kg and phorate in 3 samples at low concentration of 0.011 mg/kg to 0.019 mg/kg. In one sample, ethion is detected at concentration 0.27 mg/kg. Imazalil accounted for the highest proportion (66.6%) of pesticides detected in fruits. In 8 fruit samples, pesticide residues exceeded the maximal allowable concentration (MAC) and were labeled as unsafe for human use. Results are shown in Table 2.

### 3.2. Risk Assessment

The risk was assessed separately for each pesticide detected positive. Risk assessment was calculated according to the EFSA for acute and chronic exposure to pesticides for which the acute reference dose (ARfD) has been established. Risk assessment was performed separately for adults and for children under 6 years of age. The results are shown in Table 2.

Imazalil was the most frequently detected pesticide in our study. Imazalil showed the highest ARfD percentage of adults (max% ARfD 251%), and these values were especially high in risk assessment for children, where they amounted up to max% ARfD 1087%. Risk assessment for adults detected that imazalil values exceeded MAC in 8 (44.4%) out of 18 samples where imazalil was found, while for children, they exceeded MAC in 72.2% (13 of 18) samples.

### 3.3. Results of Animal Testing

As shown in Table 3 at the end of the experimental period, body weight of treated animals did not differ significantly from that in the control group. Unlike body weight, weight gain showed a statistically significant difference (*p* ≤ 0.05) between the experimental and control groups of animals.

There was no statistically significant difference in RBC count and hemoglobin concentration between the imazalil exposed group and the control group (*p* ≤ 0.05). However, a statistically significant (*p* ≤ 0.05) increase in WBC count was recorded in the experimental group as compared with a control group of animals (Table 4).

**Figure 1:**
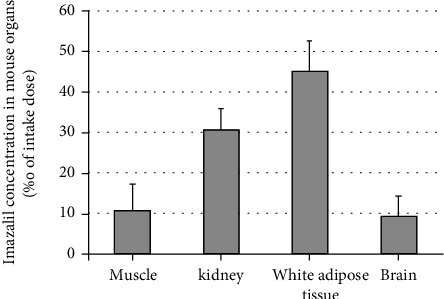
Imazalil accumulated in analyzed organs of Swiss albino mice (pooled samples of 8 mice, 4 male + 4 females). The columns represent mean values (‰) of imazalil in the muscle, kidney, white adipose tissue, and brain of Swiss albino mice after 28-day imazalil exposure. The wishers represent the standard deviation of the mean of triplicate analysis of a pooled sample.

The mean imazalil concentrations in the muscle, kidney, adipose tissue, and brain of male and female experimental animals, expressed in ‰, are shown in Figure 1. The highest imazalil levels were recorded in adipose tissue (45.2‰), followed by the kidney (30.6‰), muscle (10.6‰), and brain (9.2‰).

## 4. Discussion

In order to achieve better yields or to extend their shelf life, fruits and vegetables are treated with pesticides. Given that Croatia is a relatively small country with low agricultural production, it is forced to import fruits and vegetables from countries outside the EU. When fruits and vegetables are imported to EU member states, it is necessary to analyze pesticide residues. Fruit samples are homogenized with the peel, which often contains pesticide residues [28]. The results of the analysis show whether the sample complies with Regulation 396/2005 and whether it is safe or harmful to human health [29]. Samples that do not comply with the regulation are sent to risk assessment.

A total of 200 fruits and vegetable samples were analyzed using a gas chromatograph with mass spectrometry. The results obtained by the analysis were evaluated. The assessment of chronic and acute dietary exposure to pesticide residues is estimated by using a calculation model developed by EFSA (PRIMo-Pesticide Residues Intake Model) [30]. The most detected pesticides and pesticide with the highest ARfD percentage were imazalil. Further research was performed on laboratory mice fed with sunflower oil with added Imazalil at NOEL doses [31].

This research was conducted on fruits and vegetables that arrived at the laboratory for pesticide residue analysis. Samples were submitted by inspectors during product inspections in stores or inspection during import. The largest number of samples consisted of imported samples from countries outside EU. From 200 samples analyzed, pesticide residues were detected in 30 samples. In fruit samples, pesticide residues were found in 27 of 120 (22.5%) samples. Imazalil was detected in 18 samples at a concentration range of 0.020-4.1 mg/kg. Imazalil is a fungicide that is mainly used as a postharvest pesticide so that means that it should be located on the peel of oranges [32]. A study conducted by Swiss researchers on citrus fruits proved that imazalil was detected in 70% of cases [33]. Chlorpyriphos was found in 8 samples at a concentration ranging from 0.030 mg/kg to 0.27 mg/kg. Chlorpyriphos is an organophosphorus insecticide which is widely used in agriculture. Due to the risk to human health that this pesticide presents and also pollutes the environment, it is banned in the EU [34].

Phorate was detected in 3 samples at very low concentration from 0.011 mg/kg to 0.019 mg/kg. Phorate is organophosphate used as insecticide, and it is banned in EU. Although it is EU forbidden in third countries, it can still be found. The presence of phorate was shown in a study by Indian researchers [35]. In one sample, ethion is detected at concentration of 0.27 mg/kg.

The results of the analysis were processed and sent for risk assessment in the Croatian Agency for Agriculture and Food. The risk was assessed separately for each pesticide detected positive. Risk assessment was calculated acute and chronic exposure to pesticides for which the acute reference dose (ARfD) has been established. Risk assessment was performed separately for adults and for children under 6 years of age, and the results are shown in Table 3. The highest ARfD for the adult and children was calculated for pesticide imazalil in orange with quantified concentration of 4.1 mg/kg.

After imazalil proved to be the most common pesticide in the analyzed samples, a study was conducted on the laboratory mice. The pesticide was administered orally *via* cannula, suspended in pure edible sunflower oil before application. Control group animals received pure edible sunflower oil, 0.2 ml *per* day. Testing included measurement of body weight and weight gain, peripheral blood analysis, and determination of imazalil concentration in the muscle, kidney, adipose tissue, and brain of treated animals. Results show difference in gain weight, and an increase in WBC count was recorded in the experimental group as compared with a control group of animals (Table 4). The highest imazalil levels were recorded in adipose tissue (45.2‰) which proves tendency to accumulate.

## 5. Conclusion

Pesticide residues were detected in 30 of 200 (15.0%) fresh fruit and vegetable samples analyzed. In fruit samples, pesticide residues were found in 27 of 120 (22.5%) samples.

In 9 fruit samples, pesticide residues exceeded the maximal allowable concentration (MAC) and were labeled as unsafe for human use. Study results revealed imazalil to be the most frequently detected pesticide.

Assessment of exposure to all pesticides detected that imazalil yielded the highest percentage of ARfD value, which was most pronounced in children, even up to 1087% ARfD.

In animal experiments, imazalil exerted a high inflammatory potential and caused leukocytosis. Upon administration of NOAEL dose of imazalil for 28 days as well as after the pesticide biotransformation in the mouse body, imazalil residues were verified in all the four organs analyzed. In addition, imazalil showed the highest potential of bioaccumulation in the adipose tissue of both male and female animals, while the lowest pesticide concentrations were recorded in the brain tissue.

These results indicate bioaccumulation of the pesticide in body organs, even with the use of low pesticide doses over a longer period and following its biotransformation in the body, whereby each particular organ exhibits a variable bioaccumulation potential.

Study results indicate that imazalil poses a major risk not only due to its high prevalence but also for the high risk to human health in countries like Croatia, where great amounts of citrus fruits are consumed and citrus rind is an important dietary component.

## Figures and Tables

**Table 1:** Validation results: mean recovery and relative standard deviation from three representative samples (apple, cucumber, and lemon) inoculated with 0.1 *μ*g/ml to 0.25 *μ*g/ml.

Pesticide	Apple (*n* = 5)^∗^		Cucumber (*n* = 5)^∗^		Lemon (*n* = 5)^∗^	
	Recovery (%)	RSD (%)	Recovery (%)	RSD (%)	Recovery (%)	RSD (%)
Aldrin	98	3.6	97	1.5	104	1.2
Bromophos ethyl	91	6.8	108	5.6	102	5.9
Chlorfenvinphos	77	5.8	88	4.8	103	4.9
Chlorpyriphos	87	1.8	109	1.6	99	1.3
Chlorpyriphos-methyl	102	7.8	93	7.9	105	7.4
DDD	88	4.8	77	4.6	103	4.5
DDE	106	4.9	95	5.8	99	4.4
DDT	100	12.3	88	13.8	104	12.8
Diazinon	94	7.8	94	8.7	100	8.8
Dieldrin	104	5.6	86	7.4	102	7.5
Dichlorvos	97	2.9	90	2.7	96	2.9
Dimethoate	78	10.1	92	5.9	102	4.3
Disulfoton	103	11.7	91	10.4	104	8.1
Endosulfan-I	90	3.9	89	5.2	102	3.8
Endosulfan-II	88	2.4	96	3.6	99	1.2
Endosulfan-sulfate	91	4.6	89	2.5	102	3.7
Endrin	104	8.5	86	8.6	106	6.2
Ethion	99	7.1	89	6.2	87	6.0
HCH-alpha	104	1.9	93	2.8	109	2.8
HCH-beta	102	14.2	102	13.6	99	16.5
HCH-gamma	99	10.9	89	10.3	86	11.4
Heptachlor	99	5.4	91	5.2	104	5.7
Fensulfothion	99	12.3	84	16.4	82	17.8
Heptachlor-epoxide	99	8.4	96	4.3	109	4.5
Fosfamidon	101	4.6	102	4.8	100	5.4
Imazalil	98	8.1	98	8.4	109	6.5
Fenchlorphos	106	7.3	91	7.6	109	6.9
Fenthion	105	7.7	94	7.3	90	8.9
Malathion	92	2.8	72	2.9	100	3.3
Methacrifos	85	4.6	79	4.3	100	1.6
Methamidophos	105	16.8	94	12.6	92	19.6
Mevinphos	107	4.2	91	7.5	110	6.5
Parathion-ethyl	97	2.1	106	2.3	101	3.5
Parathion-methyl	74	11.2	76	11.4	83	11.8
Pirimiphos	100	10.7	99	10.8	106	12.8
Phenitrotion	84	4.8	106	4.3	105	4.1
Phorate	106	5.6	96	3.9	98	3.5
Quinalphos	106	8.0	90	7.8	102	7.0

^∗^Each representative sample was prepared and analyzed five times.

**Table 2:** Pesticide residue concentration and assessment of acute exposure of adults and children to detected pesticides.

Pesticide	Matrix	Concentration (mg/kg)	% of acute ARfD adults	IESTI (*μ*g/kg bw/day)	% of acute ARfD children	IESTI (*μ*g/kg bw/day)
Imazalil	Orange	4.10	251	126	1087	544
Imazalil	Orange	3.50	215	107	928	464
Imazalil	Orange	3.30	202	101	875	438
Imazalil	Orange	3.33	204	102	883	442
Imazalil	Orange	2.90	178	89	769	385
Imazalil	Orange	2.55	156	78	676	338
Imazalil	Orange	2.55	156	78	676	338
Imazalil	Orange	0.90	55	28	239	119
Imazalil	Orange	0.72	44	22	191	95
Imazalil	Orange	0.096	6	2,9	25	13
Imazalil	Clementine orange	2.40	86	43	285	142
Imazalil	Pomelo fruit	0.02	0,7	0,36	2	1,2
Imazalil	Mandarin orange	2.40	86	43	285	142
Imazalil	Mandora fruit	3.80	136	68	451	225
Imazalil	Lemon	1.53	27	14	105	52
Imazalil	Lemon	0.33	6	3	23	11
Imazalil	Grapefruit	0.37	13	6,6	58	29
Imazalil	Grapefruit	0.14	5	2,6	23	11
Ethion	Orange	0.27	41	0,83	179	3,6
Chlorpyriphos	Orange	0.22	135	6,7	584	28
Chlorpyriphos	Grapefruit	0.21	75	3,8	330	16
Chlorpyriphos	Grapefruit	0.09	32	1,6	141	7,1
Chlorpyriphos	Lemon	0.08	14	0,72	55	2,7
Chlorpyriphos	Grapefruit	0.11	39	2,0	173	8,6
Chlorpyriphos	Pear	0.03	18	0,92	83	4,2
Chlorpyriphos	Peach	0.27	101	5,1	513	26
Chlorpyriphos	Grapes	0.07	47	2,4	102	5,1
Phorate	Potato	0.014	14	0,42	72	2,2
Phorate	Tomato	0.019	10	0,30	37	1,1
Phorate	Tomato	0.011	6	0,17	21	0,64

IESTI = international estimated short-term intake; bw = body weight; ARfD = acute reference dose.

**Table 3:** Body weight and weight gain in Swiss albino mice after 28 days of treatment with imazalil expressed as mean standard deviation (SD) and median.

Group^∗^		Initial body weight at the beginning of experiment	Weight gain after 28 days of experiment
Control	Mean ± standard deviation (SD)	25.8 ± 3.3	+2.0 ± 0.8
	Median	25.2	+2.2

Imazalil	Mean ± standard deviation (SD)	25.0 ± 3.1	+0.3 ± 1.3^a^
	Median	25.5	+0.2

^∗^8 Swiss albino mice per group (4 males and 4 females). ^a^Means with superscript (letter) are significantly different from control (*p* ≤ 0.05).

**Table 4:** Number of erythrocytes (RBC), white blood cells (WBC), and hemoglobin concentration in Swiss albino mice after 28 days of treatment with imazalil expressed as mean standard deviation (SD), median, minimum, and maximum.

	Mean ± standard deviation (SD)	Median	Minimum	Maximum	Mean ± standard deviation (SD) (males + females)
Group^∗^	RBC				
Control					
Males (M)	7.98 ± 0.35	8.04	7.40	8.28	8.00 ± 1.12
Females (F)	8.01 ± 1.55	8.12	5.44	10.12	
Imazalil					
Males (M)	8.72 ± 0.79	8.40	7.80	9.80	8.89 ± 0.80
Females (F)	9.09 ± 0.84	8.90	7.84	10.24	

Group	Hemoglobin concentration				
Control					
Males (M)	108.24 ± 6.55	108.40	100.40	116.00	119.24 ± 16.41
Females (F)	128.40 ± 16.81	126.60	110.80	150.40	
Imazalil					
Males (M)	123.60 ± 13.77	124.40	100.40	142.80	128.74 ± 13.87
Females (F)	134.73 ± 12.41	135.20	113.60	150.40	

Group	WBC				
Control					
Males (M)	3.28 ± 0.37	3.20	2.94	3.79	3.24 ± 1.09
Females (F)	4.12 ± 1.73	4.52	0.92	6.00	
Imazalil					
Males (M)	3.69 ± 0.96^a^	3.60	2.22	4.88	4.16 ± 1.16^a^
Females (F)	4.56 ± 1.07^a^	4.84	2.91	6.00	

^∗^8 Swiss albino mice per group (4 males and 4 females). ^a^Means with superscript (letter) are significantly different from control (*p* ≤ 0.05).

## Data Availability

The underlying data supporting the results of the study can be found at the University of Osijek through hyperlink: http://zpio.unios.hr/wp-content/uploads/radovi/dokt.disert/gordana.jurak.pdf.
